# Response to the Boucher et al. Comments on Fleury et al. Sun Exposure and Its Effects on Human Health: Mechanisms through Which Sun Exposure Could Reduce the Risk of Developing Obesity and Cardiometabolic Dysfunction. *Int. J. Environ. Res. Public Health* 2016, *13*, 999

**DOI:** 10.3390/ijerph13121257

**Published:** 2016-12-18

**Authors:** Naomi Fleury, Sian Geldenhuys, Shelley Gorman

**Affiliations:** Telethon Kids Institute, University of Western Australia, P.O. Box 855, Perth 6872, Australia; Naomi.Fleury@telethonkids.org.au (N.F.); ms.geldenhuys@gmail.com (S.G.)

We thank Boucher et al. [1] for their interest in our recently published review [2]. 

To directly answer their query [1] regarding calcium homeostasis in our published studies [3], we observed no effect on circulating calcium after feeding male C57BL/6J mice high or low fat diets supplemented (or not) with vitamin D for 12 weeks ([3], see Figure 5C). However, chronic exposure of mice fed low (but not high) fat diets that were not supplemented with vitamin D to erythemal or sub-erythemal ultraviolet radiation (UVR), significantly reduced circulating calcium levels ([3], see Figure 5C) after 12 weeks of treatment. We are uncertain of the biological effect of these decreases in circulating calcium as reduced weight gain was observed in mice exposed to erythemal doses, but not in those exposed to sub-erythemal doses of UVR ([3], see Figure 2). However, it is important to note that circulating 25-hydroxyvitamin D (25(OH)D) levels remained low (<20 nmol/L) for all male mice fed a low fat diet that was not supplemented with vitamin D, regardless of whether or not they were exposed to UVR ([3], see Supplementary Figure 2D). This inability for male mice to increase their circulating 25(OH)D levels may be because male mice have reduced epidermal stores of 7-dehydrocholesterol [4,5] relative to female mice, and vitamin D-deficient male mice have increased renal levels of the vitamin D breakdown enzyme, 24-hydroyxlase (CYP24A1) [4] relative to vitamin D-replete male mice. 

Regarding circulating parathyroid hormone (PTH) levels, these were not detectable (<5 pg/mL, not shown) in the serum of male C57BL/6J mice from the experiments described in [3], as measured using a standard mouse-specific immunoassay (antibodies-online.com, Atlanta, GA, USA). PTH levels were also <5 pg/mL in the serum of BALB/c male mice fed the same low fat diet that was supplemented (or not) with vitamin D (not shown). It is possible that the increased calcium levels (1%–2%) in our diets (designed to maintain calcium homeostasis) suppressed circulating PTH, as observed by others [6]. It is important to note that we did not observe a significant effect of treatment with ongoing UVR on circulating 25(OH)D, calcium or PTH levels in mice fed a high fat diet [3]. The UVR treatments suppressed weight gain and signs of type-2 diabetes that were induced by feeding mice the high fat diet [3], suggesting that the effects of UVR were independent of circulating 25(OH)D and calcium. We did observe some modest effects of vitamin D supplementation on the extent of liver steatosis induced by feeding mice a high fat diet, suggesting there may be beneficial effects of dietary vitamin D similar to those referenced by [1]. However, UVR had far more potent effects than vitamin D supplementation in our animal experiments.
